# BRD4 amplification facilitates an oncogenic gene expression program in high-grade serous ovarian cancer and confers sensitivity to BET inhibitors

**DOI:** 10.1371/journal.pone.0200826

**Published:** 2018-07-23

**Authors:** Garrett W. Rhyasen, Yi Yao, Jingwen Zhang, Austin Dulak, Lillian Castriotta, Kelly Jacques, Wei Zhao, Farzin Gharahdaghi, Maureen M. Hattersley, Paul D. Lyne, Edwin Clark, Michael Zinda, Stephen E. Fawell, Gordon B. Mills, Huawei Chen

**Affiliations:** 1 Bioscience, Oncology, IMED Biotech Unit, AstraZeneca, Boston, Massachusetts, United States of America; 2 Department of Systems Biology, The University of Texas MD Anderson Cancer Center, Houston, Texas, United States of America; Universite du Quebec a Trois-Rivieres, CANADA

## Abstract

BRD4 is a transcriptional co-activator functioning to recruit regulatory complexes to acetylated chromatin. A subset of High-grade Serous Ovarian Cancer (HGSOC) patients are typified by focal, recurrent BRD4 gene amplifications. Despite previously described cancer dependencies, it is unclear whether BRD4 amplification events are oncogenic in HGSOC. We find that physiologically relevant levels of expression of BRD4 isoforms in non-transformed ovarian cells result in cellular transformation. Transcriptional profiling of BRD4-transformed ovarian cells, and BRD4-amplified HGSOC patient samples revealed shared expression patterns, including enriched MYC, and E2F1 gene signatures. Furthermore, we demonstrate that a novel BET inhibitor, AZD5153, is highly active in BRD4-amplified patient derived xenografts and uncover Neuregulin-1 as a novel BRD4 effector. Experiments involving Neuregulin-1 inhibition and exogenous addition, demonstrate Neuregulin-1 as necessary and sufficient for BRD4-mediated transformation. This study demonstrates the oncogenic potential of BRD4 amplification in cancer and establishes BRD4-amplified HGSOC as a potential patient population that could benefit from BET inhibitors.

## Introduction

Bromodomain-containing protein 4 (BRD4) is a member of the bromodomain and extraterminal (BET) family of chromatin reader proteins, which also includes BRD2, BRD3, and BRDT. BET proteins feature two conserved N-terminal bromodomains that serve to interact with N-acetyl lysine residues on histones and nuclear proteins [1–4]. BRD4 localizes to discrete genomic regions via interactions with acetylated chromatin, and BRD4 functions to regulate RNA-pol II-mediated elongation and transcription through direct interaction with the Mediator complex and pTEFb [5, 6]. By interacting directly with acetylated transcription factors, including RelA, ERα, p53, and TWIST, BRD4 can function to maintain oncogenic gene expression in cancer [7–9]. At enhancer and promoter regions, BRD4 facilitates the combinatorial interactions among acetylated histones, transcription factors, and nuclear proteins to promote cell-type specific transcription. Although BRD4 lacks catalytic activity, BET bromodomains are amenable to drug targeting by selective acetyl-lysine mimetic small-molecules. The first characterized BET bromodomain chemical probes, JQ1 and I-BET have demonstrated pre-clinical activity primarily in hematologic cancers [10–12]. Building on compelling preclinical efficacy, efforts in translating BRD4 probe compounds into clinical drug candidates have resulted in a number of ongoing clinical programs, testing BRD4 inhibition in a wide range of solid and hematologic malignancies including Nut Midline Carcinoma, Acute Myeloid Leukemia, Myelodysplastic Syndromes, Multiple Myeloma, Diffuse Large B-cell Lymphoma, and Glioblastoma Multiforme [13–21].

Epithelial ovarian cancer is the fifth most common cause of cancer-related mortality in women and the most lethal gynecologic malignancy in the United States [22]. Advances in chemotherapeutic and surgical strategies, improved understanding of natural history, and the elucidation of genetic determinants of disease have resulted in meaningful improvements in patient survival without substantially improving cure rates. Of the five epithelial histological subtypes, high-grade serous ovarian carcinoma (HGSOC) is the most malignant form of epithelial ovarian cancer and accounts for approximately 70% of all ovarian cancer cases and deaths. Despite recent advances, approximately 25% of HGSOC patients relapse within 6 months of completing platinum-taxane chemotherapy [23]. Genomic data from The Cancer Genome Atlas (TCGA) have revealed nearly ubiquitous TP53 mutations in HGSOC. In addition, somatic and/or germline BRCA1/2 mutations occur in approximately 22% of tumors and play a critical role in disease progression and therapeutic response [24] [25]. Although other recurrent oncogenic mutations in this tumor type are extremely rare, somatic copy-number alterations and whole genome duplications occur frequently in HGSOC [25, 26]. For example, frequent, recurrent, focal gene amplification has been reported in well-characterized oncogenes, such as PIK3CA, MYC, and CCNE1; however, the majority of genes within focal amplification events remain uncharacterized for their oncogenic activity, and thus potential for therapeutic intervention. For instance, a distinct ovarian cancer molecular subtype with poor prognosis has been characterized by somatic, focal copy-number amplifications on chromosome 19 [27]. Interestingly, BRD4 resides on chromosome 19p and its focal amplification has been previously reported [28, 29]. Additionally, in treatment naïve HGSOC, BRD4 amplification correlates with poor clinical outcomes [30]. However, prior to this study, the oncogenic activity imparted through BRD4 amplification in HGSOC remained uncharacterized.

Neuregulin 1 (NRG1) is a glycoprotein and tropic factor containing an epidermal growth factor (EGF)-like domain that signals by stimulating ErbB receptor tyrosine kinases. The NRG1 gene contains distinct 5’ flanking regulatory elements, which allows for six distinct proteins with at least 31 known isoforms [31]. The EGF-like domain is located on the extracellular domain and is necessary and sufficient for activation of ErbB receptor tyrosine kinases. As it relates to HGSOC patients, NRG1 has been shown to drive proliferation of ovarian cancer cells in the context of activated ErbB3/NRG1 autocrine signaling [32].

In this study, we hypothesized that focal BRD4 amplification is oncogenic, and therapeutically actionable in HGSOC patients. We sought to interrogate the functional activity of BRD4 amplification by assessing BRD4-mediated oncogenic potential in non-transformed, immortalized ovarian epithelial cells. We demonstrate the potent transforming activity of BRD4 in this setting, and show that NRG1 functions as a critical effector in BRD4-mediated cellular transformation. Our BRD4 transformation model revealed that NRG1 expression is regulated by BRD4 activity that is mediated by the SWI/SNF nucleosome remodeling complex. We also demonstrate the effectiveness of targeting BRD4 in BRD4-amplified HGSOC patient-derived xenografts and the impact on NRG1 expression using AZD5153, a bivalent BET bromodomain inhibitor that has recently entered clinical testing (NCT03205176). Finally, we demonstrate the effectiveness of co-targeting of BRD4 and NRG1 signaling in the context of BRD4-amplified patient-derived tumor models.

## Materials and methods

### ChIP-qPCR

Cells (5×10^7^) were treated with 200 nM AZD5153 for 4 hours and cross-linked with 1% formaldehyde (Sigma-Aldrich, #F8775) for 15 min at room temperature followed by 5 min treatment with 0.125 M Glycine. The cells were washed with ice-cold PBS and cell pellet was collected. The nuclear fraction was extracted by first resuspending the pellet in 10 mL of LB1 buffer (50 mM HEPES-KOH, pH 7.5, 140 mM NaCl, 1 mM EDTA, 10% glycerol, 0.5% NP-40, and 0.25% Triton X-100) for 10 min at 4°C. Cells were pelleted, resuspended in 10 mL of LB2 buffer (10 mM Tris-HCl, pH 8.0, 200 mM NaCl, 1 mM EDTA, and 0.5 mM EGTA), and mixed at 4°C for 10 min. Cells were pelleted and resuspended in 1.5 mL of sonication buffer (10 mM Tris-HCl, pH 8.0, 100 mM NaCl, 1 mM EDTA, 1 mM EGTA, 0.1% Na-deoxycholate, 1% Triton X-100 and 0.1% SDS) and sonicated in a waterbath sonicator (Diagenode Bioruptor) (50x30s pulse, medium power). The chromatin was precipitated with 80 μL of anti-FLAG M2 Magnetic Beads (Sigma-Aldrich #M8823) or 8 μg of pre-conjugated anti-IgG/Protein A Dynabeads (Invitrogen #10002D) per 100 μg of chromatin in a volume of 600 μL of sonication buffer for 4 hr at 4°C. The precipitates were washed three times with sonication buffer, once with sonication buffer containing 500 mM NaCl, once with LiCl buffer (20 mM Tris-HCl pH 8.0, 1 mM EDTA, 250 mM LiCl, 0.5% Na-Deoxycholate and 0.5% NP-40) and once with TBS (20 mM Tris-HCl pH 7.6, 50 mM NaCl, 1 mM EDTA). Precipitates were eluted in 50 mM Tris-HCl pH 8.0, 1% SDS and 1 mM EDTA, decrosslinked, RNaseA and Proteinase K digested and purified using Qiagen PCR purification kits. Quantitative real-time RT-PCR was performed with gene specific primers (Table 1) and 2xSYBR Green Master Mix (Thermo Fisher Scientific, #4385610) on ABI Prism 7900HT instrument. Percentage enrichment over the IgG control was calculated using ΔΔct method. Human negative control set I from Active Motif (#71001) was used as a negative control.

**Table 1:** List of qPCR primers.

Primers	Sequence
Myc-NR-F	tcctgggtaggaaccagttg
Myc-NR-R	actcaccaagagctcctcca
Myc-E-F	gaaatgtgagggcacatcgt
Myc-E-R	atacctgctggagcatttgg
Myc-TSS-F	ggtcggacattcctgcttta
Myc-TSS-R	gatatgcggtccctactcca
NRG1-TSS-F	tgagaaaccccacattcacg
NRG1-TSS-R	attactgcagccgctgaaag
NRG1-TSS-F	gcgcaacctagcatctttaagg
NRG1-TSS-R	gcacgcaggcgaaaataaac
NRG1-E1-F	agcagcctcgtgttcttctc
NRG1-E1-R	atggacttggccagagagaaag
NRG1-E2-F	agcctcgtgttcttctcagac
NRG1-E2-R	tgaagtcacgcaatgctgag

### Immunoprecipitation

5X10^6^ IOSE cells were lysed in lysis buffer (50 mM Tris-HCl pH 7.4, 150 mM NaCl, 1 mM EDTA, 0.5% NP-50) supplemented with protease and phosphatase inhibitors. Cell lysates were sonicated on Diagenode bioruptor for 6 cycles (30s on, 60s off) on medium setting. After sonication, cell lysates were cleared by centrifugation and then pre-cleared with Protein A magnetic beads (Life Technologies #10002D) for 1 hr at 4°C with agitation. For FLAG pull downs, pre-cleared lysates were incubated with FLAG magnetic beads (Thermo Fisher Scientific #M8823) at 4°C overnight with agitation. For immunoprecipitations with BRD4, BRD4 antibody (Abcam #ab128874) was conjugated to protein A magnetic beads using 2 mM BS reagent (Thermo Fisher Scientific #21585) in conjugation buffer (30 mM Sodium Phosphate, 0.15 M NaCl) for 30 min at RT. Conjugation reaction was stopped by quenching the reaction with 1 M Tris-HCl (pH 7.5, 50 mM final concentration). Pre-cleared lysates and BRD4 antibody-conjugated protein A beads were then incubated together at 4°C overnight with agitation. The next day, beads were washed 4 times with lysis buffer before sample buffer was added to elute the immuno-complexes. The following antibodies were used for immunoprecipitation experiments: Cell Signaling Technology: SMARCA4 (#49360), SMARCC1 (#11956), SMARCC2 (#12760), CDK9 (#2316); from Abcam: SMARCD2 (#ab81622); from Bethyl: SMARCA2 (#A301-015); SMARCB1 (#A301-087); SMARCD1 (#A301-593).

### Quantitative PCR

Total RNA was isolated using Qiagen RNeasy Kit (#74104) following manufacturer’s instructions. 2 μg RNA was converted to cDNA with High-Capacity cDNA Reverse Transcription Kit (Applied Biosystems #4368814). qPCR reaction was set up with TaqMan^®^ Gene Expression Master Mix (Thermo Fisher Scientific #4369016) and carried out on an Applied Biosystems 7500 HT instrument. TaqMan gene expression probes are from Thermo Fisher Scientific, NRG1 (#HS00247620), MYC (#HS00905030), SMARCCA1 (#HS00268265), SMARCA4 (#HS00231324), GAPDH (#HS02758991), and Actin (#HS00984230). The amplified transcript level of each specific gene was normalized to that of GAPDH.

Gene copy-number was measured using TaqMan^®^ Copy-number Assay kits following manufacturer’s instructions (Thermo Fisher Scientific #4403326 and #4403316).

### Cell lines and reagents

Immortalized ovarian surface epithelial cells (IOSE) were purchased from Applied Biological Materials Inc within 6 months, and cultured in Prigrow I medium containing 10% FBS according to the manufacturer’s instructions. IOSE cells were transduced with lentivirus containing pTRIPZ-empty vector, BRD4 long isoform, or BRD4 short isoform. Cells were selected with and maintained on 0.175 μg /mL puromycin. Antibodies used for western blot analysis were from Cell Signaling Technology (Danvers, MA): anti-GAPDH (#2118), anti-Histone H3 (#4499), anti-pERBB2 Y1222/1221 (#2243), anti-ERBB2 (#3250), anti-pAKT S473 (#9271), anti-AKT (#9272), anti-pERK1/2 T202/Y204 (#9201), anti-ERK1/2 (#9102); from Abcam (Cambridge, MA): anti-BRD4 (#ab128874); and from R&D Systems (Minneapolis, MN): anti-NRG1 (#MAB377).

### Mass spectrometry

#### Immunoprecipitation

IOSE cells (2 × 10^7^) were washed with ice-cold PBS and cell pellet was collected. The nuclear fraction was obtained from cells using reagents from the Universal Magnetic Co-IP Kit (Active Motif, Carlsbad, CA, #54002) according to the manufacturer’s instructions. 40 μL anti-FLAG M2 magnetic beads (Sigma-Aldrich, #8823) were added to nuclear lysates containing 1 mg of total protein for each immunoprecipitation reaction. Immunoprecipitation procedure was carried out according to the manufacturer’s instructions. The beads were washed ten times in 1 mL of RIPA buffer and twice in 100 mM ammonium hydrogen carbonate (AMBIC) solution.

#### Enzymatic digestion

On-bead digestion was performed by incubating samples at 56°C under gentle mixing for 30 minutes with 60 μL of 10 mM dithiothreitol in Triethylammonium bicarbonate (TEAB). Then 10 μL 150 mM iodoacetamide in 10 mM TEAB was added followed by incubation at room temperature for 30 minutes. Subsequently, 10 μL of trypsin in 50 mM TEAB (Total 1–2 μg) was added. Samples were allowed to digest under gentle mixing overnight at 37°C; the digestion was quenched by adding 5 μL of 10% formic acid. Tryptic digest samples were centrifuged at 12,000–16,000 × *g* for 5 minutes and the supernatants were pooled and dried in a vacuum centrifuge. Digested samples were desalted using ZipTipC18 (Millipore, Billerica, MA) based on the recommended procedure by the manufacturer. The eluted peptides were vacuum dried.

#### Mass spectrometric analysis

Peptide mixture was reconstituted in 15 μL of 0.1% aqueous formic acid. Each sample was injected (10 μL) by using Easy nLCII (Thermo Fisher Scientific) and loaded using a pre-column (Easy Column C18, 5 μm,100 μm ID, Thermo Fisher Scientific) and separated on the PicoFrit column (ProteoPep column C18, 360 μm OD x 50 μm ID, Tip 10 μm ID, New Objective) with a piecewise linear gradient of acetonitrile containing 0.1% formic acid at flow rate at 300 nL/min over 240 minutes. This HPLC system was coupled on-line to Orbitrap mass spectrometer (Velos, Thermo Fisher Scientific), using a nano ESI (Nanospray Flex Ion Source, Thermo Fisher Scientific). The mass spectrometer was running in data dependent mode with the one full MS1 scan (400–1650 m/z) in Orbitrap at resolution setting of 60,000, followed by top twenty ions data-dependent MS2 scan in the linear trap.

#### Database search

Thermo Scientific Proteome Discoverer software version 1.4 was used to search MS/MS spectra against the Swiss-Prot human database (http://www.uniprot.org/downloads) using SEQUEST HT search engine. Static modifications included carbamidomethylation (C) and Dynamic modifications included methionine oxidation and deamidation of Asparagine and Glutamine. The mass tolerances of the precursor ion and the fragment ions were set to 10 ppm and 0.6 Da, respectively. Full tryptic specificity was required with up to two miss cleavage Sites. Resulting peptide hits were filtered for maximum 1% FDR using the Percolator algorithm and with the minimum number of two peptides per protein.

### Animals and patient-derived tumor models

Female NSG mice aged 5 to 6 weeks were obtained from Jackson Laboratories (Bar Harbor, ME) and maintained under specific-pathogen-free conditions in an AAALAC-accredited facility. Animal protocols were approved by the AstraZeneca R&D Boston Institutional Animal Care and Use committee. All animal work was conducted in accordance with ARRIVE guidelines [33].

Tumor fragments from high grade serous ovarian PDX models OV0857F, OV2022F and OV0452F were purchased from Jackson Labs, (Sacramento, CA). Details of the Jackson Lab models can be found at www.JAX.org. PDX model HOXF-062 of serous papillary ovarian was established at AstraZeneca using a surgical biopsy from Maine Medical Center (Portland, ME) obtained with patient consent and Institutional Review Board approval. Tissue size of ~ 8 mm^3^ was implanted subcutaneously with a trocar in the right flank of mice. The mice were randomized into groups of 8 to 10, based on tumor volume and treated with either vehicle or AZD5153 at 5 or 10 mg/kg QD. AZD5153 was formulated in 0.5% HPMC/0.1% Tween80 and dosed orally 20 to 32 days after tumor fragment implantation. Tumor length and width were measured by caliper and tumor volume calculated using the formula volume = width^2^* length *0.52. Mice were anesthetized with isoflurane inhalation prior to and during subcutaneous implantation of tumor fragments. Animals were monitored during implantation and allowed to recover before being returned to clean cages.

### Soft agar assay

1 mL (1x10^4^) IOSE cells were plated on top of a 1.6% agarose base layer in 1.0% low melting agarose in triplicate in 24-well plate. After 3 weeks, cells were stained for 24 hr with iodonitrotetrazolium chloride at a final concentration of 0.5 mg/mL. Colonies were visualized and counted using a Gelcount^™^ (Oxford Optronix) software version 1.1.8.0.

### Proliferation assays

1x10^3^ cells were seeded per well in triplicate onto a 96-well glass bottom plate (Greinier, #655892) and incubated at 37°C, 5% CO2 for 6 days. Confluence was measured hourly using an IncuCyte ZOOM^®^ Live-content imaging system with ZOOM Controller Version 2013A (Essen Bioscience). Data was analyzed using IncuCyte analysis software.

### Immunoblotting

IOSE cells were lysed with 1xSDS lysis buffer (60 mM Tris-HCl pH 6.8, 1% SDS, and 10% glycerol). For PDX tumors, tissues were homogenized in 10 mL of lysis buffer (20 mM Tris-HCl pH 8.0, 150 mM NaCI, 1% NP-40) supplement with 100 μL of Phosphatase Inhibitor Cocktail2 (Sigma-Aldrich #P5726), 100 μL of Phosphatase Inhibitor Cocktail3 (Sigma -Aldrich #P0044), 1 tablet of complete protease inhibitor cocktail (Roche, #11836153001). 20 μg of total protein were loaded for western blots analysis.

### RNA-seq

For cell line samples, 10 days post-transduction 1x10^6^ cells per line was snap frozen using ethanol and dry ice. For PDX samples, 50 mg of PDX Tumors treated with AZD5153 or vehicle were snap frozen in liquid nitrogen. Experiments were performed in biological triplicate. RNA isolation, cDNA library generation, and sequencing to 12M (for cells) or 25M (for tumors) reads on the Illumina HighSeq was carried out at Q^2^ Solutions (http://www.q2labsolutions.com/). Differential expression calling was performed via edgeR in R using the exactTest method. Signal-to-noise (S2N) ranking was used for gene set enrichment analysis (GSEA) input. A significance threshold of log2FC +/- 1 and FDR < 0.05 was used.

### shRNA and siRNA-mediated protein knockdown

shRNA lentiviral viral particles targeting NRG1 (#SHCLND-NM_004495) and SMARCA4 (#SHCLNV-NM_003072) were obtained from Sigma-Aldrich. IOSE cells were transduced with targeting lentiviral particles and then selected for 10 days in 1.0 mg/mL G418. For siRNA knockdown, cells were seeded into 6-well plates at a density of 1 × 10^5^ cells/well. Twenty-four hours later, cells were transfected with 25 pmol siRNA against SMARCA4 (Dharmacon #L010431), or siRNA negative control (Invitrogen #AM4611) using Lipofectamine RNAiMAX (Invitrogen #13778075) according to the manufacturer’s instructions.

### JQ1 cancer cell line pharmacology screening

Sanger cancer cell line pharmacology screen and data analysis were performed as previously described [34].

### Flow cytometry cell cycle analysis

Cell cycle analysis of BRD4-expressing IOSE cells was performed by staining cells with BrdU-APC and 7-AAD using a BD Pharmingen APC BrdU Flow Kit (#552598) as per the manufacturer’s instructions on a FACSCanto flow cytometer. Analysis and gating was performed using FlowJo software.

### Statistical analysis

Results are depicted as the mean ± standard error of the mean. Statistical analyses were performed using Student’s *t*-test (* P<0.05; ** P<0.01; *** P<0.001; **** P<0.0001). GraphPad Prism (v6, GraphPad) was used for statistical analysis.

## Results

### BRD4 mRNA and protein expression is correlated with BRD4 copy-number in high-grade serous ovarian cancer

**Fig 1:**
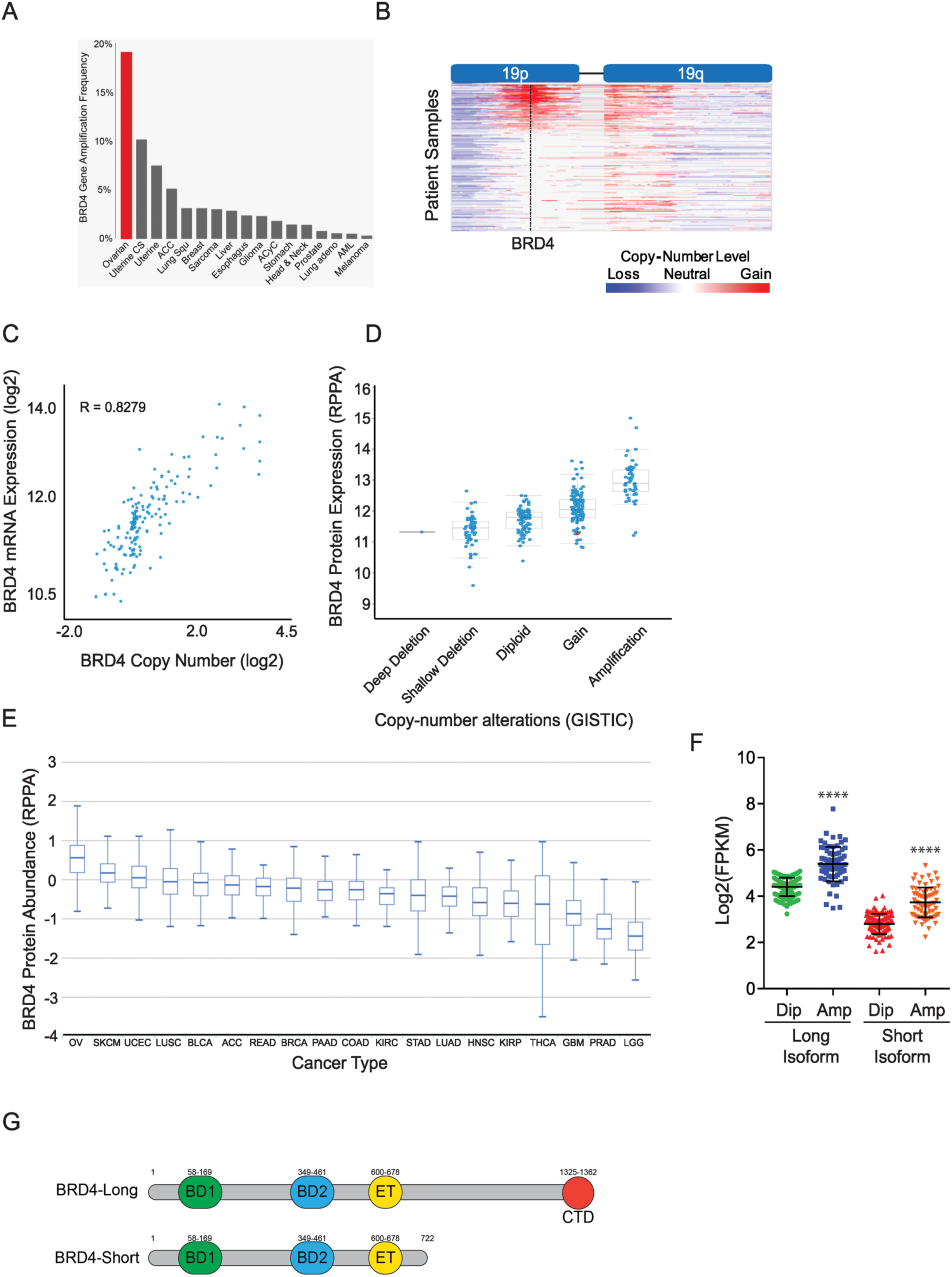
BRD4 is focally amplified in ovarian serous adenocarcinoma. **(A)** BRD4 amplification frequency was surveyed from 559 SNP6.0 Affymetrix copy-number arrays from primary tumors represented in TCGA. **(B)** IGViewer showing copy-number gain across chromosome 19 in 559 primary high-grade serous ovarian patient samples. **(C)** Analysis of BRD4 mRNA expression plotted against BRD4 copy-number from high-grade serous ovarian patient samples from TCGA. (**D**) BRD4 protein expression via RPPA correlated with BRD4 gene copy-number status. (**E**) Ovarian cancer exhibits highest levels of BRD4 protein expression via RPPA. **(F)** Analysis of BRD4 mRNA isoforms and copy-number from high-grade serous ovarian patient samples from TCGA. (**G**) Domain organization of BRD4 long isoform and short isoform (BD1 and BD2: tandem bromodomains; ET: extra-terminal domain; CTD: C terminal domain).

We utilized The Cancer Genome Atlas (TCGA) data to interrogate BRD4 amplification across all represented cancer subtypes. BRD4 amplification occurs most frequently (~18%) in high-grade serous ovarian cancer patients (Fig 1A). These patients exhibit focal BRD4 gain on chromosome 19p (Fig 1B). GISTIC analysis confirmed that BRD4 is the 4^th^ most frequently amplified peak in HGSOC (Data not shown). Furthermore, there was a strong correlation (R = 0.83) between DNA copy-number and BRD4 mRNA expression, suggesting selective evolutionary pressure for BRD4 gain in cancer (Fig 1C). Additionally, BRD4 copy-number correlates with BRD4 protein expression levels as measured by reverse phase protein array (RPPA) (Fig 1D). Further, of all cancer types tested for BRD4 protein expression by RPPA in cBioPortal, ovarian cancer exhibits the highest level of BRD4 protein expression (Fig 1E) [35]. To assess the functional importance of both long and short BRD4 spliced isoforms, we examined mRNA expression across HGSOC TCGA patient data. The expression level of both long and short BRD4 isoforms is significantly upregulated in a concordant fashion in BRD4-amplified HGSOC (Fig 1F). Both BRD4 isoforms share identical N-terminal protein domain architecture, where the tandem bromodomains are located, while the long isoform possesses a unique C-terminal domain (Fig 1G).

### BRD4 over-expression is sufficient to transform immortalized ovarian cells

**Fig 2:**
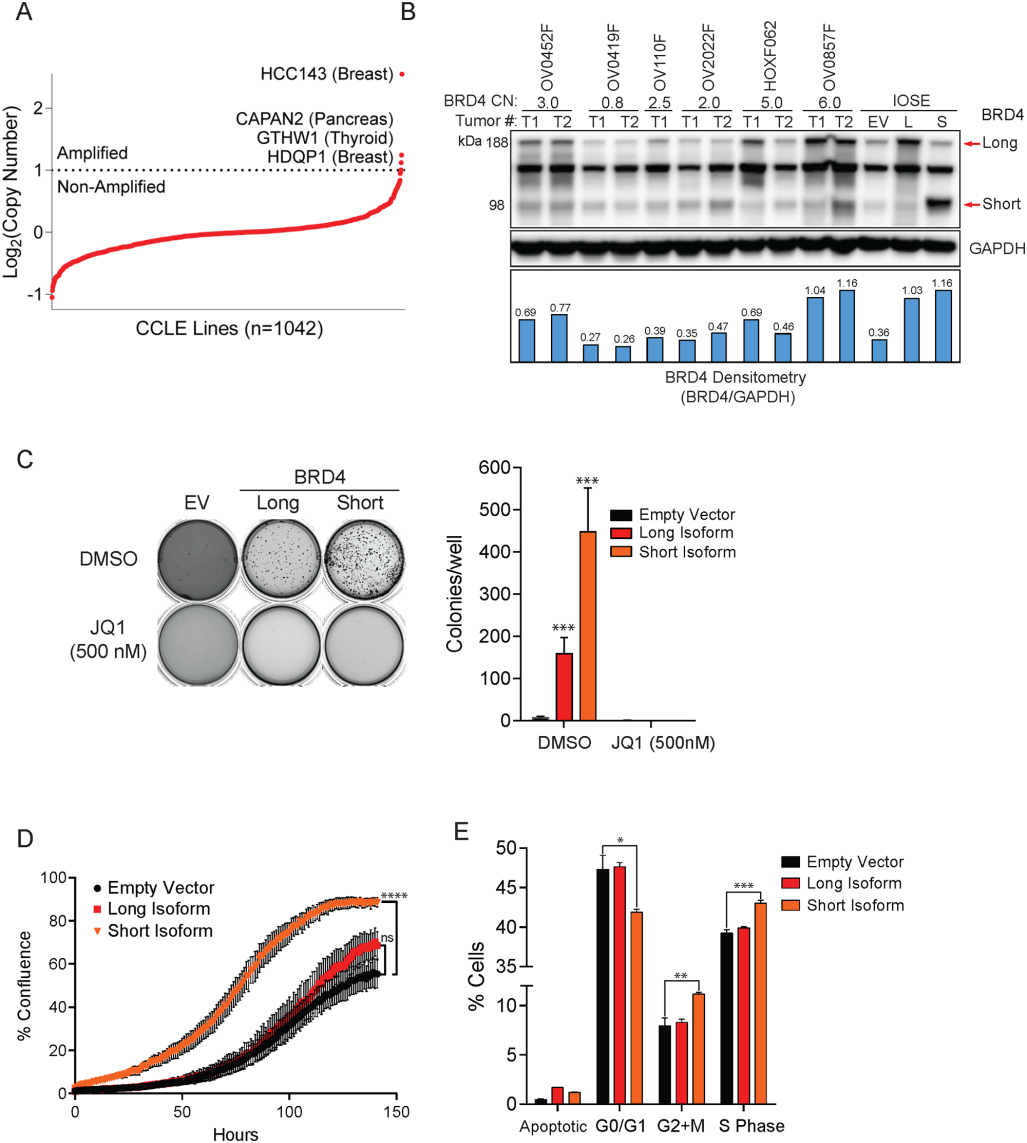
BRD4 expression is sufficient to transform immortalized ovarian surface epithelial cells. **(A)** Analysis of BRD4 copy-number from 1042 cell lines (CCLE). **(B)** BRD4 long and short-isoform expression in BRD4-transduced IOSE cells, and several BRD4-non-amplified (OV0419F, OV110F, OV2022F and OV0452F) and BRD4-amplified (HOXF062 and OV0857F) patient derived xenograft models. BRD4 copy-number (CN) in different PDX models were determined using TaqMan^®^ Copy-number Assay by qPCR. **(C)** Colony formation potential of IOSE cells expressing BRD4 -/+ 500 nM JQ1 was assessed using a soft-agar assay. **(D)** Proliferation of BRD4 expressing IOSE cells was examined by measuring cell confluence over time via live cell imaging. **(E)** Cell cycle analysis of long and short BRD4-isoform expressing IOSE cells.

To study the functional consequences of BRD4 amplification in ovarian cancer, we surveyed BRD4 copy-number across the Cancer Cell Line Encyclopedia (CCLE). Out of 1042 cell lines examined only four exhibited BRD4 amplification, as defined by GISTIC2.0 (Fig 2A). None of the four lines with amplified BRD4 were derived from ovarian cancer patients. To circumvent this challenge, we engineered a BRD4 amplified model through enforced expression of BRD4 in a non-transformed, immortalized ovarian surface epithelial cell (IOSE) background. The BRD4 expression level achieved in IOSE was similar to levels found in BRD4-amplifed HGSOC patient derived xenografts (Fig 2B).

In order to assess the oncogenic potential of BRD4 in ovarian cancer, we examined the anchorage-independent growth potential of BRD4 long and short-isoform expressing IOSE cells. Compared to empty vector transduced cells, both BRD4 long and short-isoform expressing IOSE gained robust colony formation potential, a cellular phenotype associated with oncogenic transformation (Fig 2C). The colony formation potential of short-isoform expressing IOSE was greater than long-isoform expressing counterparts, which could potentially result from more efficient ectopic protein expression of the short-form. Plating BRD4 expressing IOSE in the presence of 500 nM JQ1, a BET-bromodomain selective tool small-molecule inhibitor, abrogated colony formation potential, confirming the dependence of colony formation on BRD4 bromodomain function. Conversely, enforced expression of BRD4 was unable to confer IL3 growth independence in BaF3 cells (S1A–S1D Fig), thus demonstrating that the transforming activity of BRD4 may be cell-type specific. In order to assess the growth kinetics of BRD4 expressing IOSE cells, we assayed cell proliferation via phase-contrast live cell imaging. Short-isoform expressing IOSE achieved maximum confluence approximately 1.5–2 times faster than empty vector IOSE (Fig 2D). The increased proliferative capacity of short-isoform expressing IOSE coincided with a significant steady-state increase in S-Phase, and G2/M populations as assessed by combined BrdU and 7AAD staining by flow cytometry (Fig 2E). However, the differential between long-isoform expressing and the empty vector is not significant under these experimental conditions.

### BRD4 expressing IOSE and BRD4 amplified high-grade serous ovarian carcinoma patients have common cancer-related transcriptional features

**Fig 3:**
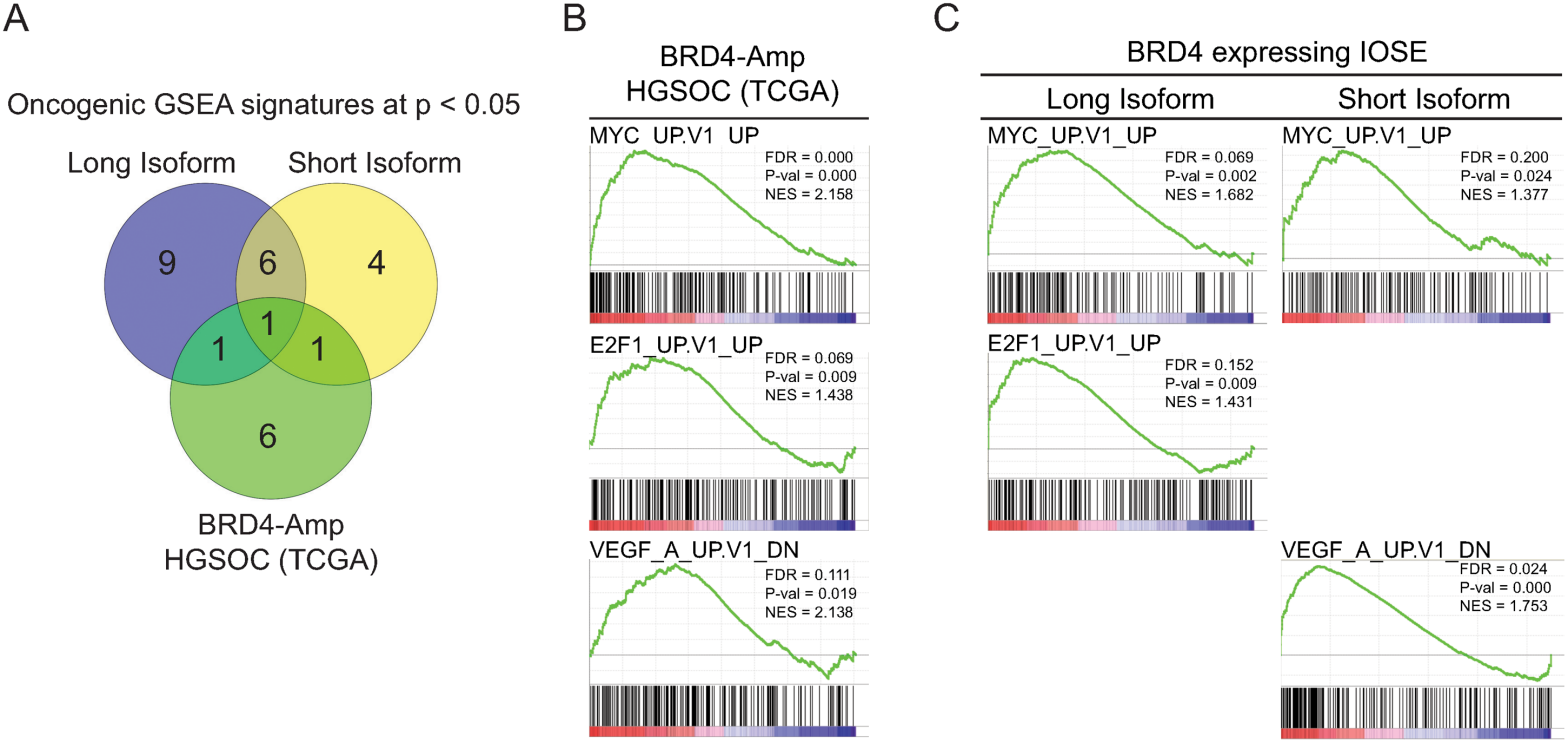
BRD4 engineered IOSE cells share oncogenic transcriptional features with BRD4-amplified high-grade serous ovarian cancer patients. **(A)** Venn diagram of oncogenic signatures enriched at p < 0.05 in BRD4-IOSE cells and HGSOC samples from TCGA. **(B-C)** Oncogenic GSEA signatures shared between BRD4-IOSE cells and HGSOC samples from TCGA.

In order to interrogate the transcriptional landscape of BRD4-amplified high-grade serous ovarian cancer (HGSOC) patients, we analyzed TCGA RNA-sequencing data. The differential expression, expressed as signal-to-noise, between BRD4-amplified tumors (GISTIC ≥ 2) and non-amplified tumors (GISTIC < 2) was used to generate gene lists and perform GSEA. We similarly performed GSEA on BRD4 long and short-isoform expressing IOSE cells. From the three GSEA lists generated, we examined shared oncogenic gene sets using a cutoff of p < 0.05 (Fig 3A; S2 Fig). Enriched MYC (FDR = 0.000, p = 0.000), E2F1 (FDR = 0.069, p = 0.009) and VEGF (FDR = 0.111, p = 0.019) signatures overlapped with either long or short-isoform expressing IOSE cells (Fig 3B and 3C). These shared oncogenic transcriptional profiles reveal significant, concordant gene expression patterns between HGSOC patients with BRD4 amplification and BRD4 overexpressing IOSE cells, suggesting the relevance of our model system to study the potential oncogenic function of BRD4 in HGSOC.

### NRG1 is a BRD4 transcriptional effector and is necessary and sufficient for IOSE transformation

**Fig 4:**
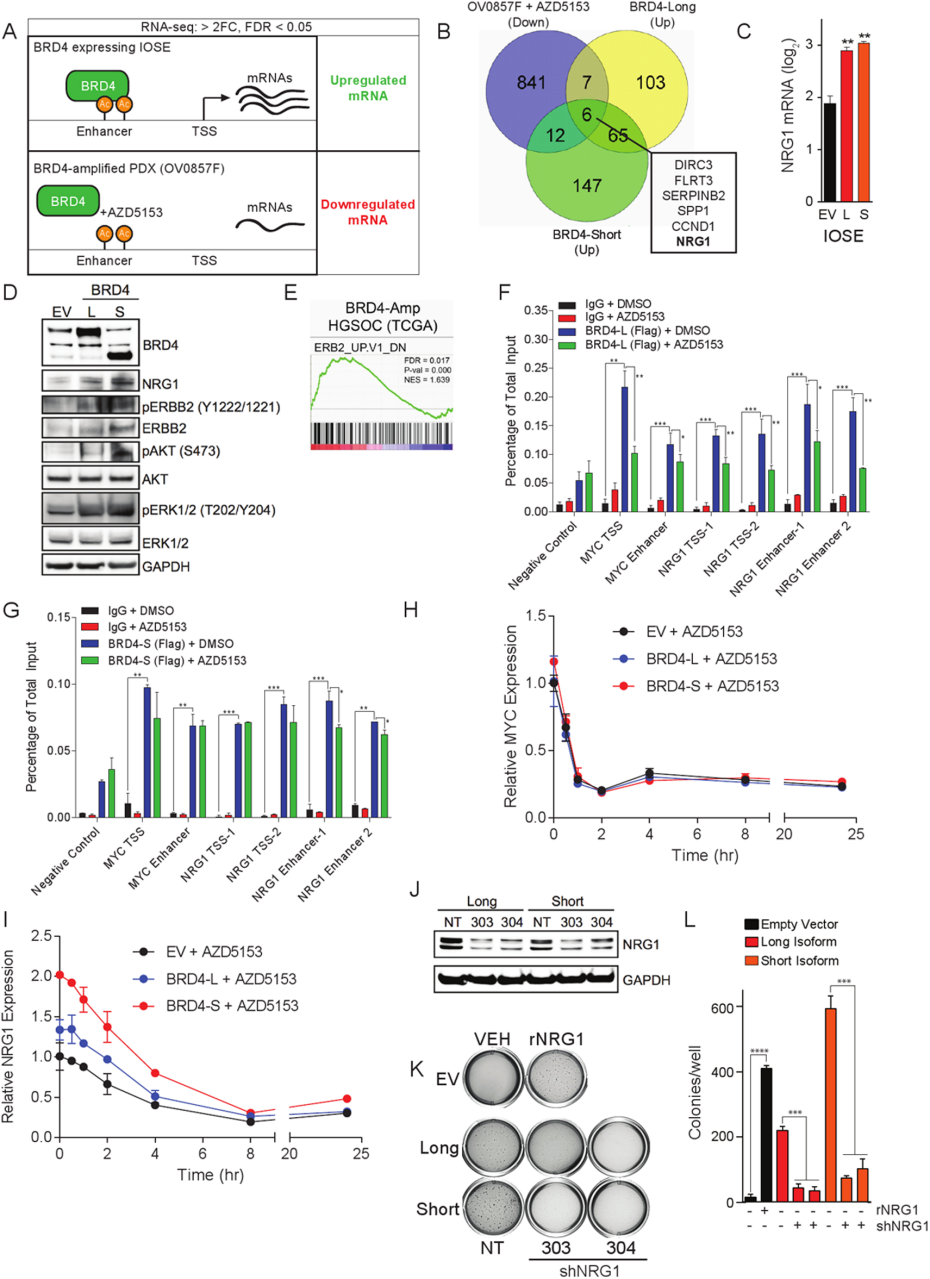
NRG1 is a critical transcriptional effector that is necessary and sufficient for BRD4-mediated transformation. **(A)** Two experimental schemes used to identify BRD4 transcriptional effectors. **(B)** Venn diagram illustrating mRNA transcripts > 2FC downregulated by AZD5153 in OV0857F tumors, and mRNA transcripts > 2FC upregulated by either short- or long-BRD4 expression in IOSE cells. **(C)** qPCR for NRG1 mRNA performed on BRD4 expressing IOSE cells. **(D)** Immunoblots confirming modulation of RTK signaling from BRD4 expressing IOSE cells. **(E)** ERBB2 GSEA profile is enriched in BRD4-amplified HGSOC TCGA patients. **(F-G)** ChIP-qPCR studies of BRD4 binding to NRG1 TSS or putative enhancers in IOSE cells expressing either the long- or short-isoform of BRD4. Cells were treated with 200 nM AZD5153 for 4 hr and ChIP was performed with antibodies against either FLAG or IgG. Percentage enrichment was calculated over total input DNA. Data represent average ± SEM of three replicates. **(H-I)** qPCR analysis of MYC and NRG1 levels in IOSE cells overexpressing BRD4 isoforms after 200 nM AZD5153 treatment for indicated periods of time. All expression levels are normalized to GAPDH and presented as fold increase relative to empty-vector expressing cells. **(J)** NRG1 knockdown confirmed by immunoblot in BRD4-expressing IOSE cells transduced with a scramble control or shNRG1 lentiviral vectors. **(K-L)** BRD4-IOSE cells expressing shNRG1 vectors were plated in soft agar to examine colony formation potential. Empty-vector IOSE cells were plated in soft agar +/- 10 ng/mL recombinant NRG1.

In order to define critical effectors in BRD4-amplified ovarian cancer, we deployed RNA-sequencing to identify genes upregulated in BRD4 long and short-isoform expressing IOSE. Similarly, we identified genes downregulated by AZD5153, a previously described potent and selective bivalent BRD4/BET inhibitor [36] in OV0857F, a BRD4-amplified PDX (Fig 4A). We examined the intersection of these three transcriptional data sets and found 6 putative BRD4 transcriptional effectors [37](Fig 4B). From the six putative effectors, NRG1 was chosen for further study because of previously described roles in supporting the in vivo proliferation and metastasis of ovarian cancer [32, 38]. In both long- and short-isoform expressing IOSE cells, NRG1 mRNA was elevated approximately two-fold over empty-vector transduced cells (Fig 4C).

We hypothesized that increased levels of NRG1 in BRD4-expressing IOSE would lead to activation of relevant downstream signaling pathways including HER2/3, Ras, PI3K and STAT3. To test this hypothesis, we deployed a phospho-RTK protein array to examine phosphorylation signaling events following induction of BRD4 expression. Consistent with the known signaling mechanism of NRG1, we observed increased pAKT (S473), pErbB2 (pan-Y), pErbB3 (pan-Y), pERK1/2 (T202/Y204), and pSTAT3 (Y705) in BRD4 expressing IOSE as compared to empty vector IOSE (S3 Fig). We performed immunoblots to confirm activation of ErbB signaling and observed increases in levels of pErbB2, pAKT, and pERK1/2 in IOSE cells expressing BRD4 (Fig 4D).

Based on signaling data from our BRD4 engineered system, we hypothesized that BRD4-amplified HGSOC patients would exhibit similar dysregulated signaling patterns. In support of this hypothesis, GSEA performed on HGSOC TCGA RNA-sequencing data revealed an enriched (FDR = 0.017, p = 0.000) ERBB2 gene signature associated with BRD4-amplified HGSOC patient tumors (Fig 4E).

In order to examine whether BRD4 could mediate NRG1 transcription through direct promoter and enhancer loading we performed ChIP experiments. As shown in Fig 4F and 4G, BRD4 is enriched at MYC transcription start site (TSS) and enhancer regions. BRD4 is displaced from MYC TSS and enhancer regions through BET inhibition via AZD5153, consistent with the previously reported relationship between BRD4 and MYC [37]. Similar to the MYC loci, BRD4 binding was detected at two independent NRG1 promoter and enhancer sites; the addition of AZD5153 to these cells prevented BRD4 loading to NRG1 promoter and enhancer regions. However, BRD4 binding to both MYC and NRG1 promoter and enhancer regions was more sensitive to BET inhibition via AZD5153 in the long-isoform expressing cells.

To test the kinetics of BET inhibitor-mediated NRG1 mRNA inhibition, we performed time course experiments in IOSE cells with AZD5153. As shown in Fig 4H and 4I, NRG1 mRNA levels are rapidly reduced following addition of 200 nM AZD5153. Although AZD5153-induced MYC mRNA modulation occurs with faster kinetics, NRG1 mRNA level was similarly depleted by 50% within 4hr; consistent with a BRD4-mediated transcriptional mechanism.

To assess whether NRG1 expression is required for BRD4-mediated IOSE transformation, we stably knocked down NRG1 using RNAi (Fig 4J). Soft agar colony formation potential of both BRD4 long and short-isoform expressing IOSE was markedly decreased via NRG1 knockdown with two independent NRG1-targeted shRNA hairpins (Fig 4K and 4L), confirming a requirement for NRG1 expression in BRD4-mediated IOSE cell transformation.

We hypothesized that the signal provided by NRG1 would be sufficient to transform IOSE in the absence of BRD4. To test this hypothesis, we performed soft-agar assays in the presence of 10 ng/mL recombinant NRG1. In empty-vector transduced IOSE, the presence of 10 ng/mL NRG1 was sufficient to induce colony formation, yielding a level similar to that of BRD4-expressing IOSE (Fig 4K and 4L).

### NRG1 suppression is a pharmacodynamic marker in BRD4-amplified ovarian cancer patient derived xenografts

**Fig 5:**
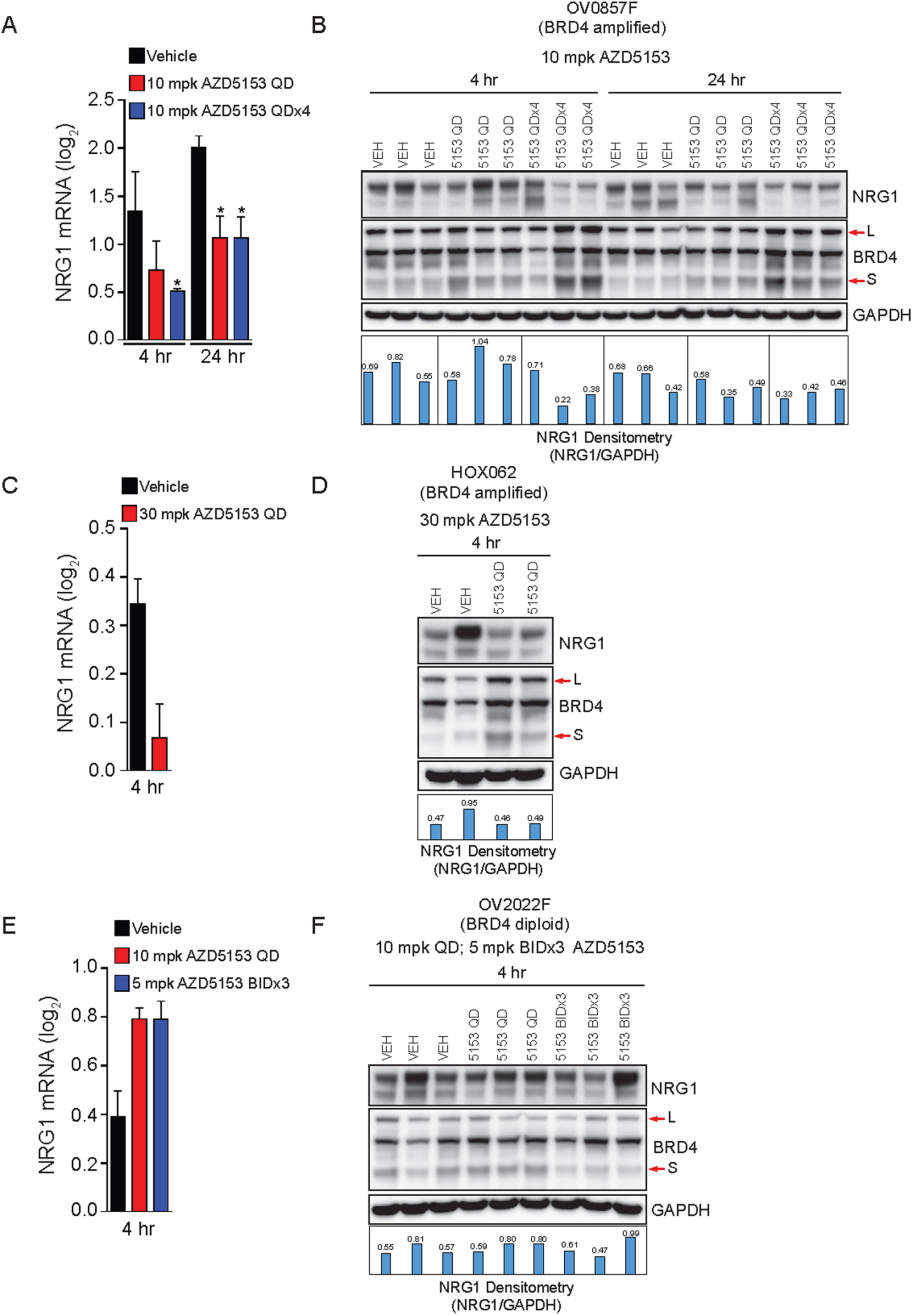
NRG1 is a pharmacodynamic marker of BRD4-amplified HGSOC patient-derived xenografts. **(A)** NRG1 mRNA expression measured using RNA-seq in OV0857F tumors at 4 hr and 24 hr following 10 mg/kg AZD5153 p.o. once (QD) or for four days (QDx4). **(B)** NRG1 protein levels measured by immunoblot in OV0857F tumors at 4 hr and 24 hr following a single or 4-repeat p.o. dose of 10 mg/kg AZD5153. **(C)** NRG1 mRNA expression measured using RNA-seq in HOXF062 tumors at 4 hr following 30 mg/kg AZD5153 p.o. (QD). **(D)** NRG1 protein levels measured by immunoblot in HOXF062 tumors at 4 hr following a single p.o. dose of 30 mg/kg AZD5153. **(E)** NRG1 mRNA measured using RNA-seq in OV2022F tumors at 4 hr following either 10 mg/kg p.o. AZD5153 QD or 5 mg/kg AZD5153 BIDx3. **(F)** NRG1 protein levels measured by immunoblot in OV2022F tumors at 4 hr following either 10 mg/kg QD or 5 mg/kg BIDx3 AZD5153.

We hypothesized that NRG1 may be a BRD4-amplification effector, and that the suppression of NRG1 expression by AZD5153 in HGSOC patient-derived xenografts might be restricted to BRD4-amplified PDX. To test this hypothesis, we screened a repository of ovarian cancer patient-derived xenografts for BRD4 copy-number (S4 Fig). We selected three patient derived xenografts for further study: OV0857F (BRD4 CN = 6), HOXF062 (BRD4 CN = 5) and OV2022F (BRD4 CN = 2). In the two BRD4-amplified PDX, OV0857F and HOXF062, a single dose of AZD5153 was sufficient to suppress NRG1 expression at the mRNA and protein level (Fig 5A–5D). In addition, we observed increased protein levels of BRD4 in response to AZD5153 in BRD4-amplified PDX models (Fig 5B and 5D). We suspect this phenomenon is due to increased BRD4 protein stabilization, as we have observed it in other cell lines and xenograft model systems (data not shown). Enhanced downregulation of NRG1, or more significant BRD4 stabilization, was observed with 4-day repeat dosing (QDx4) in OV0857F tumors. Conversely, in OV2022F lacking BRD4 gene amplification, NRG1 mRNA and protein were not significantly modulated by AZD5153, even under the more stringent BRD4 inhibition conditions of twice daily dosing for 3 days (BIDx3) (Fig 5E and 5F). Interestingly, we did not observe BRD4 protein stabilization under all treatment conditions in OV2022F model (Fig 5F).

### BRD4 amplification confers sensitivity to BET bromodomain inhibitors in HGSOC

**Fig 6:**
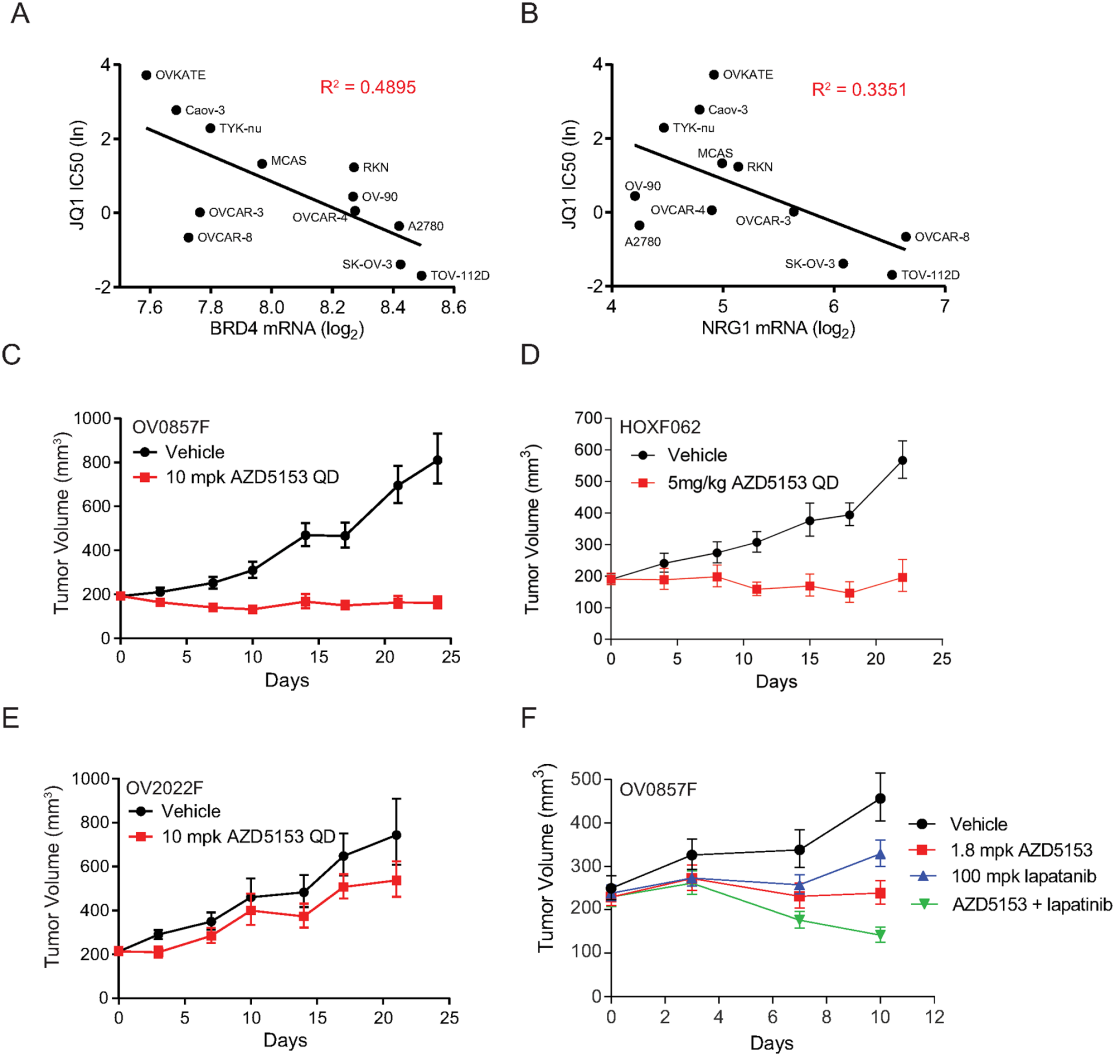
BRD4 amplification, NRG1 modulation and BET bromodomain inhibitor efficacy correlate in HGSOC. **(A)** Correlation of JQ1 IC50 and BRD4 mRNA level in 12 ovarian cancer cell lines. **(B)** Correlation of JQ1 IC50 and NRG1 mRNA in 12 ovarian cancer cell lines. **(C)** Tumor volume expressed as geometric mean for vehicle treated (n = 8) and AZD5153 treated (10 mg/kg QD, p.o.; n = 8) NSG mice implanted with OV0857F tumor fragments. **(D)** Tumor volume expressed as geometric mean for vehicle treated (n = 10) and AZD5153 treated (5 mg/kg QD, p.o.; n = 10) NSG mice implanted with HOXF062 tumor fragments. **(E)** Tumor volume expressed as geometric mean for vehicle treated (n = 7) and AZD5153 treated (10 mg/kg QD, p.o.; n = 7) NSG mice implanted with OV2022F tumor fragments. (**F**) Tumor volume expressed as geometric mean for vehicle treated (n = 9), AZD5153 treated (1.8 mg/kg, QD, p.o., n = 9), lapatinib treated (100 mg/kg, QD, p.o., n = 9), and AZD5153 and lapatinib combination treated NSG mice implanted with OV0857F tumor.

In order to assess the relationship between BRD4 amplification or expression level and sensitivity towards BRD4 inhibition, we utilized Sanger cell line screening data, which provided a 3-day JQ1 IC50 on 12 ovarian cancer cell lines [39]. In the 12 ovarian cancer cell lines tested, the level of BRD4 mRNA positively correlated with JQ1 IC50 (R^2^ = 0.49) (Fig 6A). We performed the same analysis for endometrial cell lines (n = 8) and triple negative breast cancer cell lines (n = 10), both tumor types are known to harbor BRD4 amplification; however, we did not find a similar correlation (R^2^ = 0.0863 and R^2^ = 0.002 respectively; S5A and S5B Fig). We similarly examined the relationship between NRG1 mRNA level and JQ1 IC50, and found a similar correlation (R^2^ = 0.34) (Fig 6B).

We tested the in vivo anti-tumor activity of AZD5153 on two BRD4-amplified patient-derived xenografts, OV0857F and HOXF062, as well as one non-amplified PDX OV2022F (Fig 6C–6E). In both BRD4 amplified models, over the 25-day course of drug administration, at 5 and 10 mg/kg QD, AZD5153 led to tumor stasis, with 100% tumor growth inhibition (TGI) in OV0857F and 98% TGI in HOXF062 respectively (Fig 6C and 6D). Conversely, the non-BRD4-amplified model, OV2022F, was largely refractory to AZD5153 at 10 mg/kg QD (Fig 6E). In all patient derived models with BRD4 amplification tested with AZD5153, we observed NRG1 downregulation (Fig 5A–5D). Additionally, considering BRD4 linkage to MYC has been demonstrated in multiple disease settings, we examined the anti-tumor activity of AZD5153 in a MYC amplified (but also with BRD4 copy-number gain of 3) ovarian patient-derived xenograft, OV0452F. At 5 mg/kg, AZD5153 induced a mean tumor regression of 39% (S5C Fig). Pharmacokinetic and pharmacodynamic analysis revealed robust MYC repression at 2, 4 and 8 hr following acute AZD5153 administration, supporting MYC modulation as an on-target anti-tumor mechanism in this model (S5D Fig).

To test whether inhibition of NRG1 signaling could result in anti-tumor activity in BRD4-amplified HGSOC tumors, we employed lapatinib, an orally available tyrosine kinase inhibitor with activity against the HER pathway. As shown in Fig 6F, lapatinib is active in OV0857F tumors, and the combination of lapatinib and AZD5153 yielded synergistic anti-tumor activity, resulting in tumor regression. However, the combination regimen used requires further optimization, as animal bodyweight loss in the combination group resulted in early study termination (S5E Fig). Furthermore, AZD5153 monotherapy was well tolerated at 10 mg/kg QD when administered as monotherapy in BRD4-amplified PDX models, with animals losing less than 10% body weight by day 30 (S5F Fig).

### A BRD4-SWI/SNF interaction maintains NRG1 expression and is necessary for BRD4-mediated transformation

**Fig 7:**
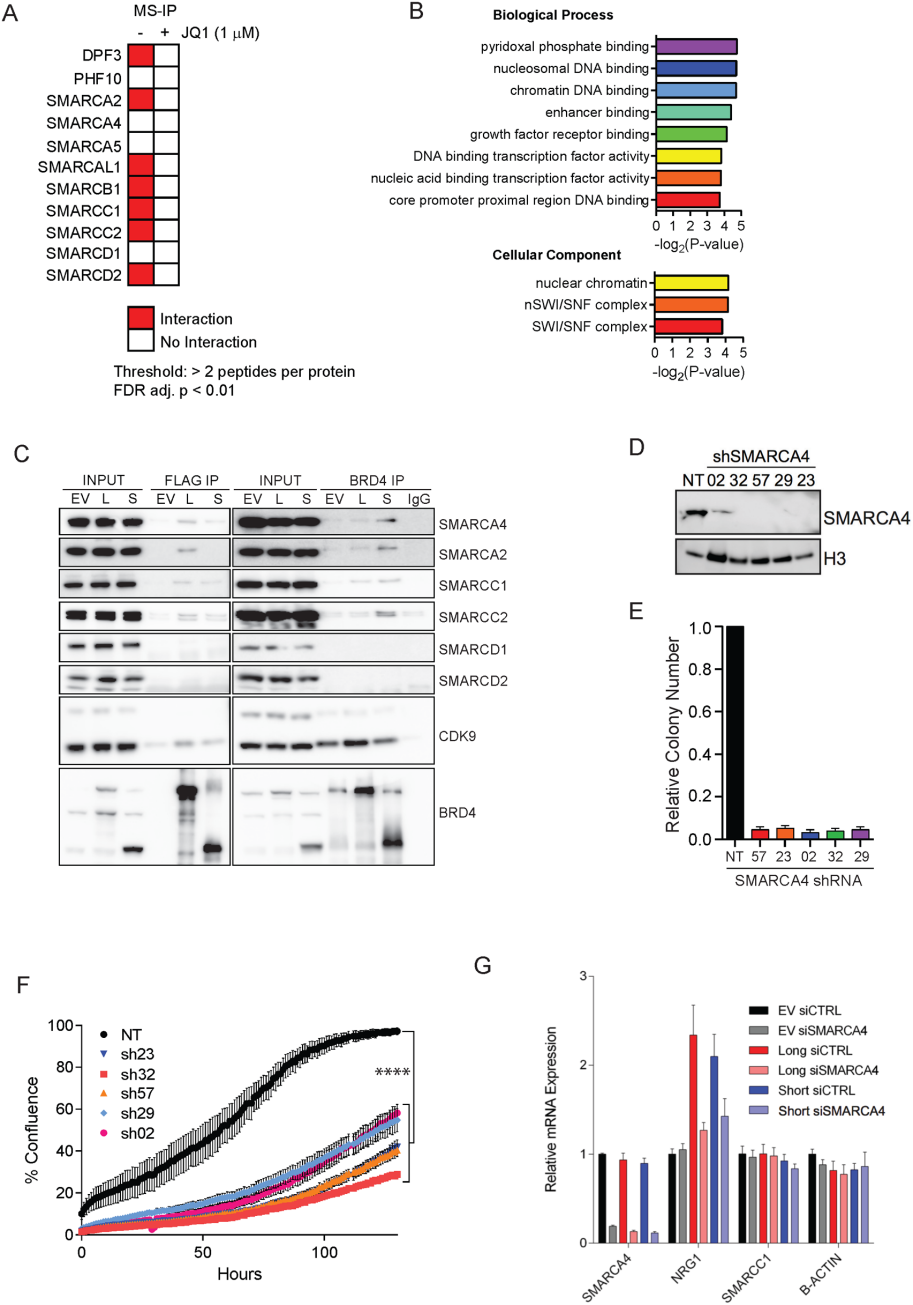
BRD4-SWI/SNF interaction maintains NRG1 expression and is necessary for BRD4-mediated transformation. **(A)** Summary of IOSE proteomics experiment. **(B)** Gene-ontology analysis of BRD4 short-isoform proteomics hits. **(C)** Immunoprecipitations were carried out using FLAG (left) or BRD4 (right) antibodies in IOSE cells expressing BRD4 long and short isoforms. Western blot analysis of the resulting immuno-complexes was carried out to determine the association between BRD4 isoforms and members of the SWI/SNF complex. CDK9, a component of the pTEFb complex that interacts with the long isoform of BRD4, is used as a positive control. **(D)** shRNA-mediated SMARCA4 knockdown in BRD4-short expressing IOSE cells. **(E)** Soft-agar colony formation of shSMARCA4 expressing BRD4 short isoform-expressing IOSE cells. **(F)** Proliferation of shSMARCA4 expressing BRD4 short isoform-expressing IOSE cells. **(G)** qPCR analysis of NRG1 levels in IOSE cells overexpressing BRD4 isoforms after 4 days of siRNA-mediated SMARCA4 knockdown.

We used proteomics as an unbiased approach to identify BRD4 protein binding partners. We included 1 μM JQ1 to assess bromodomain-specific interacting proteins. Our proteomics data revealed BRD4 interactions among 12/25 of the SWI/SNF complex members (Fig 7A). Network analysis performed by ToppGene Suite identified Biological process and Cellular Component Gene Ontology terms related to SWI/SNF function and localization significantly enriched in short-isoform proteomics hits (Fig 7B). Interestingly, SWI/SNF proteins were not detected by the mass spectrometry analysis of the pulldown of BRD4 long-form. We suspect that the lack of detection may be due to the relative low abundance of SWI/SNF proteins compared to other BRD4 long-form interacting partners and the threshold of detection sensitivity of discovery-mode proteomics. In order to validate our mass spectrometry findings, we performed immunoprecipitations, using FLAG antibody to specifically pull down either long or short isoform of BRD4. We performed immunoblots to examine association of each isoform with select SWI/SNF complex members, using CDK9 as a positive control [40]; immunoprecipitations were also carried out using a BRD4 antibody that recognizes both isoforms of BRD4 (Fig 7C). Consistent with our proteomic data, we demonstrate an interaction between short-isoform of BRD4 and SWI/SNF complex members, including SMARCA4, SMARCA2, SMARCC1, and SMARCC2. Interestingly, similar interactions were observed with BRD4 long-isoform. Thus, the lack of long-form-SWI/SNF interactions in our IP-MS dataset is likely due to technical limitations. Additionally, weaker bands were observed with empty vector (EV) control under the conditions of BRD4 IP with BRD4 antibody (Fig 7C, right panel), due to the presence of endogenously-expressed BRD4. Taken together, the data from IP-MS proteomic work and subsequent IP-western provide strong evidence supporting physical interaction of BRD4 isoforms with the SWI/SNF complex.

In order to assess functional dependence of SWI/SNF activity on BRD4-mediated transformation, we stably transduced BRD4-short-expressiong IOSE with SMARCA4 shRNA vectors (Fig 7D). shRNA-mediated SMARCA4 knockdown was sufficient to abrogate colony formation potential (Fig 7E) and proliferative capacity (Fig 7F) of BRD4-short expressing IOSE cells. To assess whether SWI/SNF activity is required for oncogenic BRD4-mediated transcriptional events, we examined expression of NRG1 following SMARCA4 knockdown. The level of NRG1 mRNA was significantly reduced upon SMARCA4 knockdown, in both long- (p<0.001) and short-isoform (p = 0.0009) expressing IOSE cells, revealing a NRG1 expression dependency on SWI/SNF activity (Fig 7G).

## Discussion

In these studies we assessed the potential oncogenic activity of recurrent, focal BRD4 amplification in high-grade serous ovarian cancer (HGSOC). We deployed IOSE as a model system to test the phenotypic consequences of enforced BRD4 isoform expression. Although previous reports had identified focal BRD4 amplification in HGSOC, our study is the first to demonstrate the transforming activity of both short and long isoforms of BRD4 in an ovarian cell background. We show that both long and short isoforms of BRD4 are coordinately expressed in BRD4-amplified HGSOC tumors, and BRD4 copy-number is correlated with protein expression. Similar to a previous study in breast cancer, the short isoform of BRD4 appeared to have more potent transforming activity than the long isoform of BRD4 [41]. The difference in transformation potential between BRD4 isoforms could potentially be explained by the presence and function of a C-terminal domain in the long isoform of BRD4, which mediates interaction between BRD4 and pTEFb. This phenomenon could also be explained by increased ectopic protein expression efficiency exhibited with the short isoform. We hypothesize that the presence of both BRD4 isoforms may be required for cooperative oncogenic activity in the context of high-grade serous ovarian cancer. However, further studies will be required to fully characterize the independent activity of each BRD4 isoform in this context. Given the absence of BRD4-amplified ovarian cancer cell lines, we used IOSE cells stably expressing the different BRD4 isoforms at levels similar to those in patients with amplified BRD4. Therefore, the IOSE model we generated represents the only reported cell line model for evaluation of the role of BRD4 in BRD4-amplified ovarian cancers. Concordant gene expression patterns between BRD4-IOSE and BRD4-amplified HGSOC served to support the biological relevance of this system.

NRG1 has been shown to contribute to proliferation, survival and metastasis of ovarian cancer cells. Our study is the first to uncover NRG1 as a transcriptional effector of BRD4. BRD4 binds to NRG1 promoter and enhancer regions in a bromodomain-dependent manner. Interestingly, our data demonstrate that cells expressing the long-isoform of BRD4 are more sensitive to BRD4 depletion at NRG1 promoter and enhancer loci, compared to short-isoform expressing cells. Isoform-specific chromatin binding properties could be mediated through differential interactions at specific loci; elucidating the differences in genome-wide chromatin binding patterns of both BRD4 isoforms will be an important avenue for future studies. Further, we demonstrate that NRG1 signaling is required for BRD4-mediated transformation, and NRG1, in the absence of enforced BRD4 expression, is sufficient to transform IOSE cells. Interrogation of BRD4-amplified HGSOC TCGA data revealed an enriched ERBB2 signature, consistent with activated NRG1 signaling in this tumor subset. Although lapatinib exhibited anti-tumor activity against BRD4-amplified tumor models, and gave significant combination activity with AZD5153, further investigation will be required to interrogate the role of NRG1 as a therapeutic target. Additional experiments that employ NRG1 neutralizing antibodies, and/or HER2/3-specific agents could provide a first step towards this end. In addition, recurrent NRG1 gene fusions have been reported in lung adenocarcinoma; NRG1-altered models may prove useful to further test the applicability of NRG1 as a therapeutic target [42, 43]. Further, it will be important to assess whether the transcription of NRG1 fusion proteins is mediated through BRD4, and whether BET bromodomain inhibitors could represent a therapeutic option for these patients.

Uncovering the interaction between SWI/SNF components and both isoforms of BRD4, as well as the functional requirement of SWI/SNF activity for BRD4-mediated transformation are additional insights from our study. The pro-tumor role for SWI/SNF defined is distinct from its role as a tumor suppressor as described previously in other cancers, including common ARID1A loss-of-function mutations in ovarian clear cell carcinoma [44–46]. Interestingly, our data demonstrate that in HGSOC, the SWI/SNF complex may function to maintain oncogenic gene expression, similar to what has been previously described in acute myeloid leukemia, where SMARCA4 is necessary to maintain oncogenic MYC expression [47]. Correspondingly, we demonstrate that SMARCA4 is necessary to maintain NRG1 transcription in IOSE cells. We speculate that maintenance of NRG1 transcription occurs through chromatin remodeling events, facilitated by interaction between both isoforms of BRD4. Future studies examining the genome-wide, isoform-specific patterns of BRD4 and SWI/SNF chromatin binding could shed additional light on the importance of BRD4-SWI/SNF interactions.

BRD4-amplified HGSOC tumors are mutually exclusive with BRCA1/2 mutations and therefore represent a tumor subtype with few therapeutic options beyond platinum-based chemotherapy. Our study demonstrates the oncogenic activity of BRD4 and positions it as an appropriate therapeutic target in this patient subset. The effectiveness of AZD5153 in BRD4-amplified patient-derived xenografts further establishes a rationale to examine the clinical effectiveness of candidate BET bromodomain inhibitors, such as AZD5153, in BRD4-amplified HGSOC.

## Supporting information

S1 Fig Enforced BRD4 expression does not confer BaF3 IL3-growth independence. (TIFF)

S2 Fig Enriched oncogenic gene signatures in BRD4-engineered IOSE cells and BRD4-amplified TCGA HGSOC patient tumors. (TIFF)

S3 Fig Relative phosphoprotein expression in BRD4-engineered IOSE cells. (TIFF)

S4 Fig Copy-number alterations in ovarian cancer patient derived xenografts. (TIFF)

S5 Fig JQ1 IC50 does not correlate with BRD4 mRNA level in endometrial cancer and triple negative breast cancer cell lines; MYC/BRD4 co-amplified PDX OV0452 is sensitive to AZD5153. (TIFF)

S6 Fig Uncropped immunoblots corresponding to cropped images in Fig 4. (TIFF)

S7 Fig Uncropped immunoblots corresponding to cropped images in Fig 7C. (TIFF)

S8 Fig Uncropped immunoblots corresponding to cropped images in Fig 7D. (TIFF)
