# Supplementary figures and images for: BRD4 amplification facilitates an oncogenic gene expression program in high-grade serous ovarian cancer and confers sensitivity to BET inhibitors
Source: PLoS One. 2018 Jul 23;13(7):e0200826. doi: 10.1371/journal.pone.0200826 (PMC6056044; doi:10.1371/journal.pone.0200826)

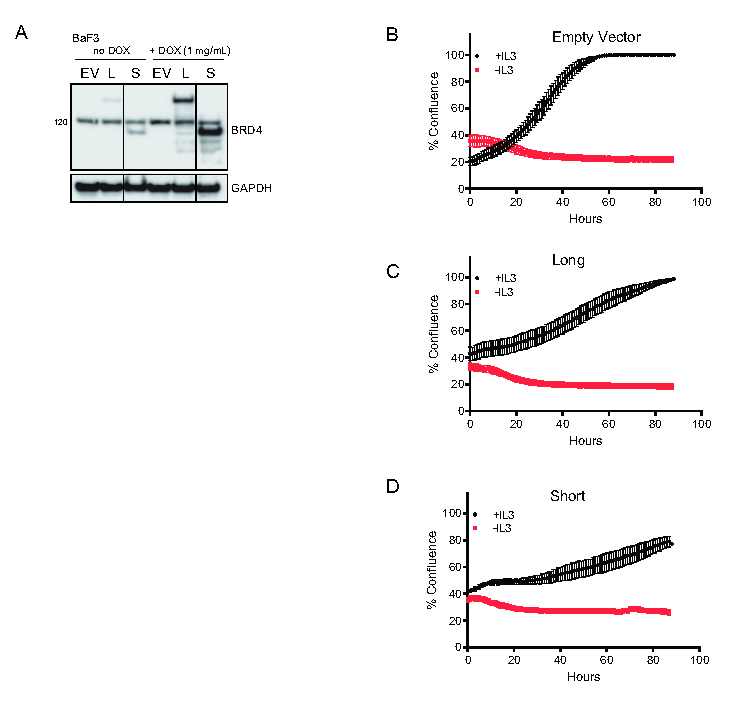

Supplement: S1 Fig — (TIFF) [file pone.0200826.s001.tiff]

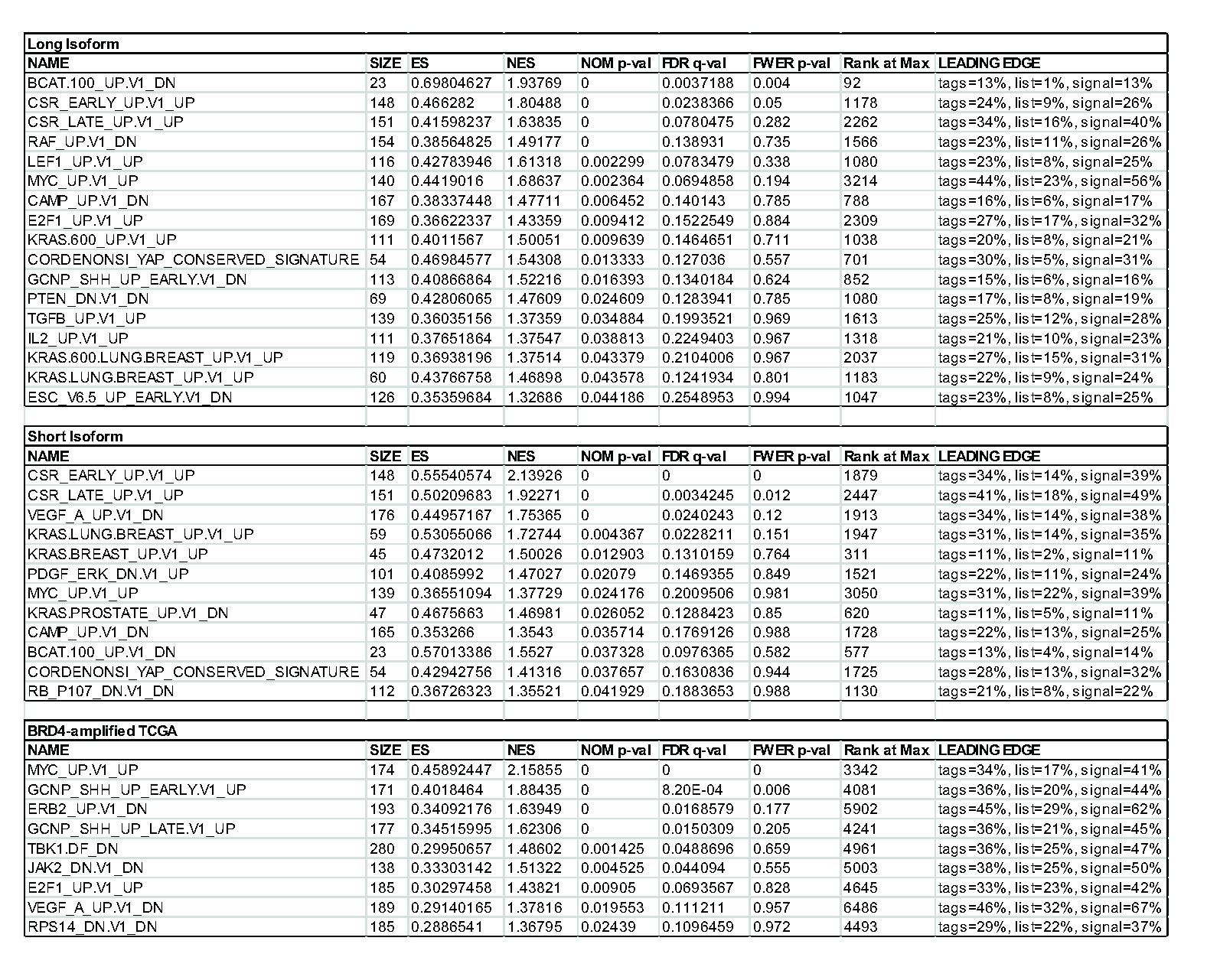

Supplement: S2 Fig — (TIFF) [file pone.0200826.s002.tiff]

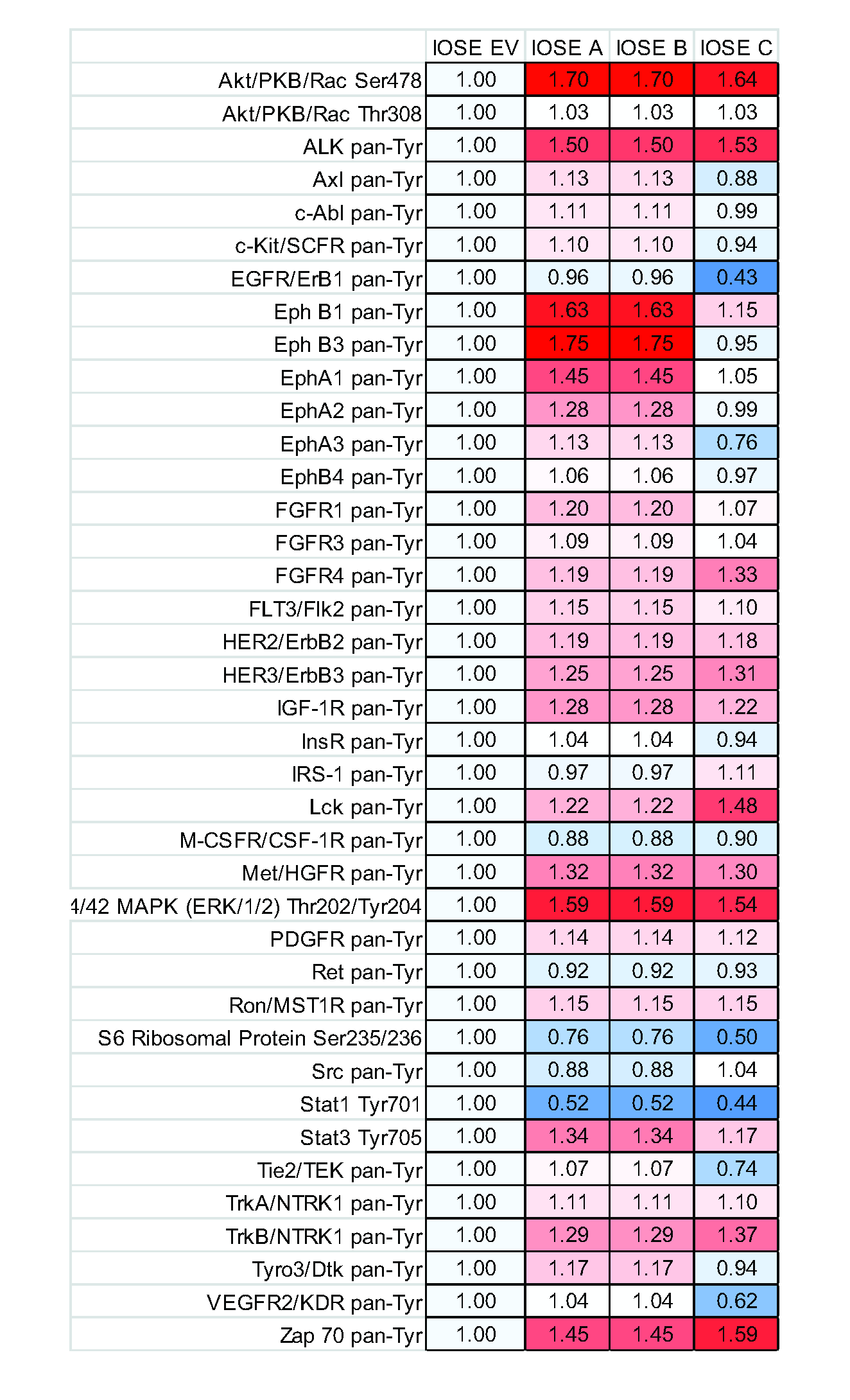

Supplement: S3 Fig — (TIFF) [file pone.0200826.s003.tiff]

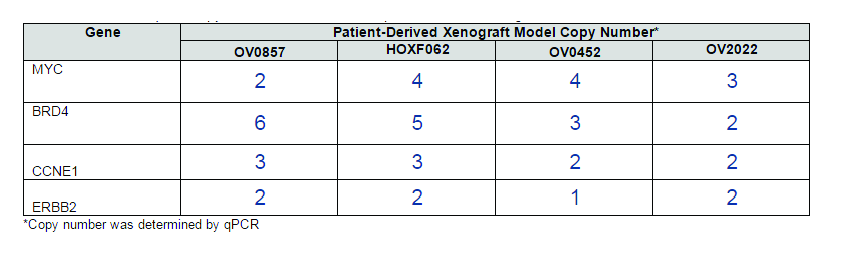

Supplement: S4 Fig — (TIFF) [file pone.0200826.s004.tiff]

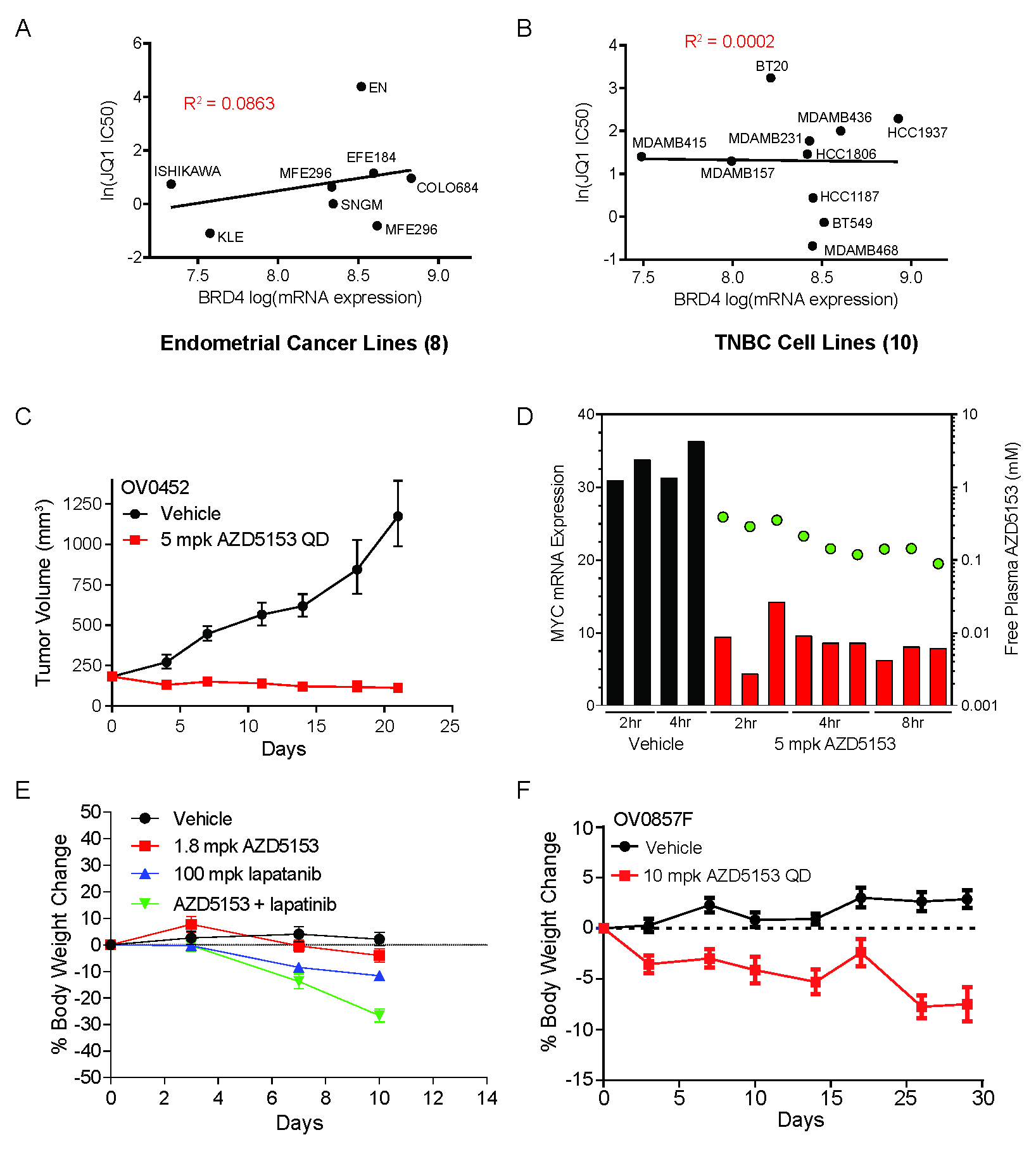

Supplement: S5 Fig — (TIFF) [file pone.0200826.s005.tiff]

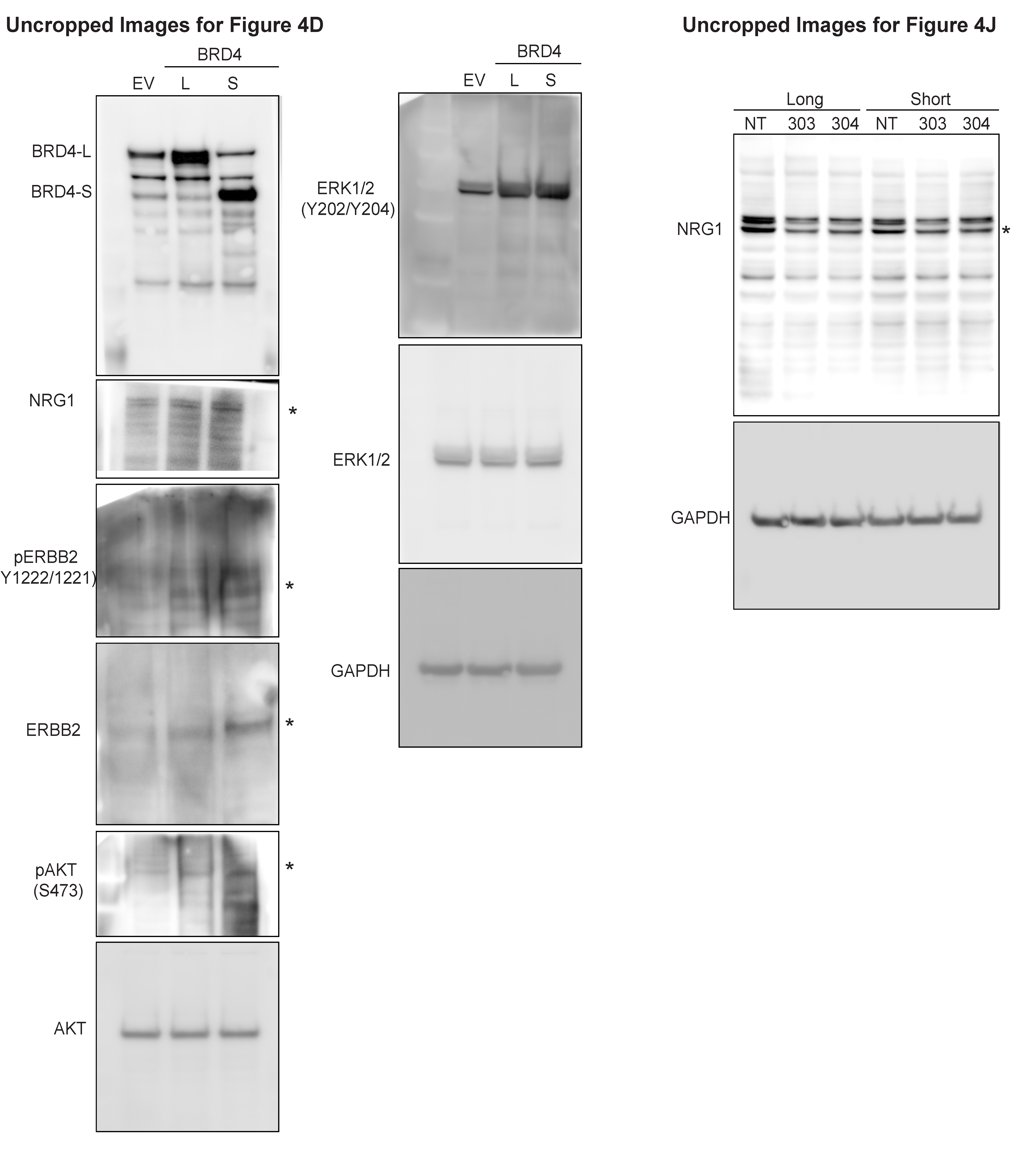

Supplement: S6 Fig — (TIFF) [file pone.0200826.s006.tiff]

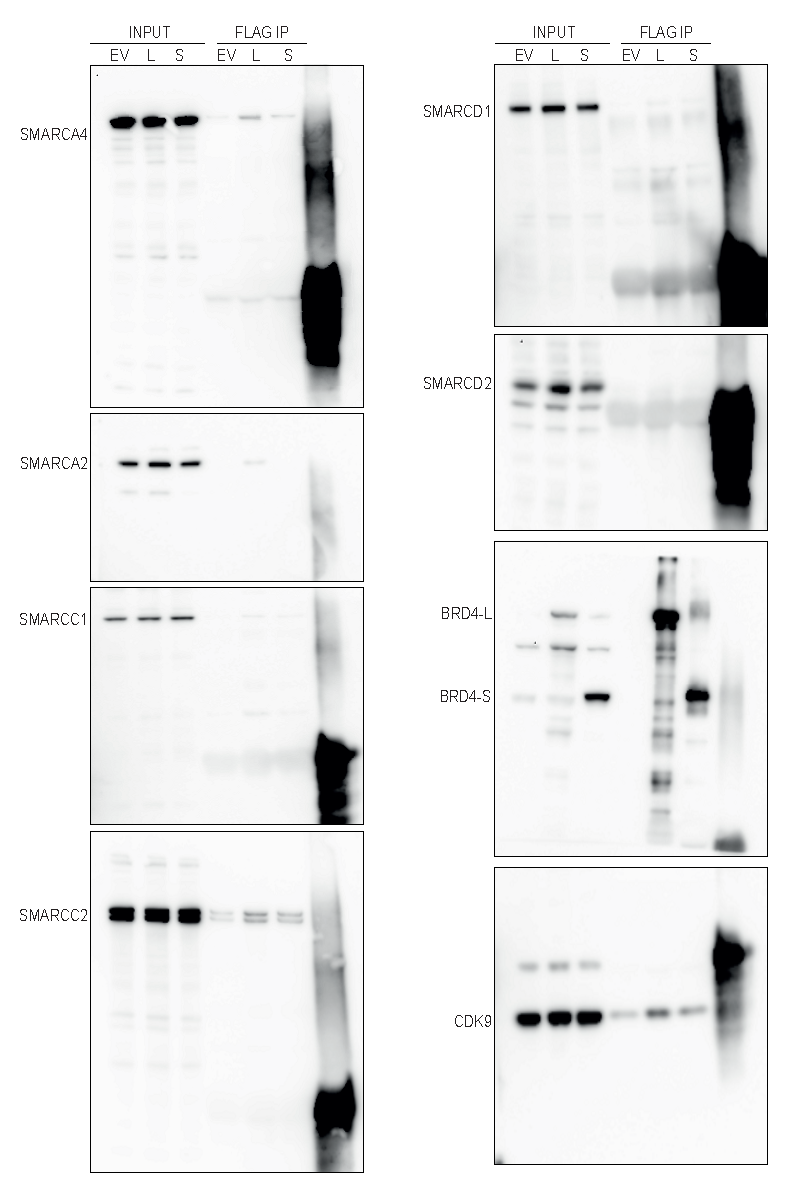

Supplement: S7 Fig — (TIFF) [file pone.0200826.s007.tiff]

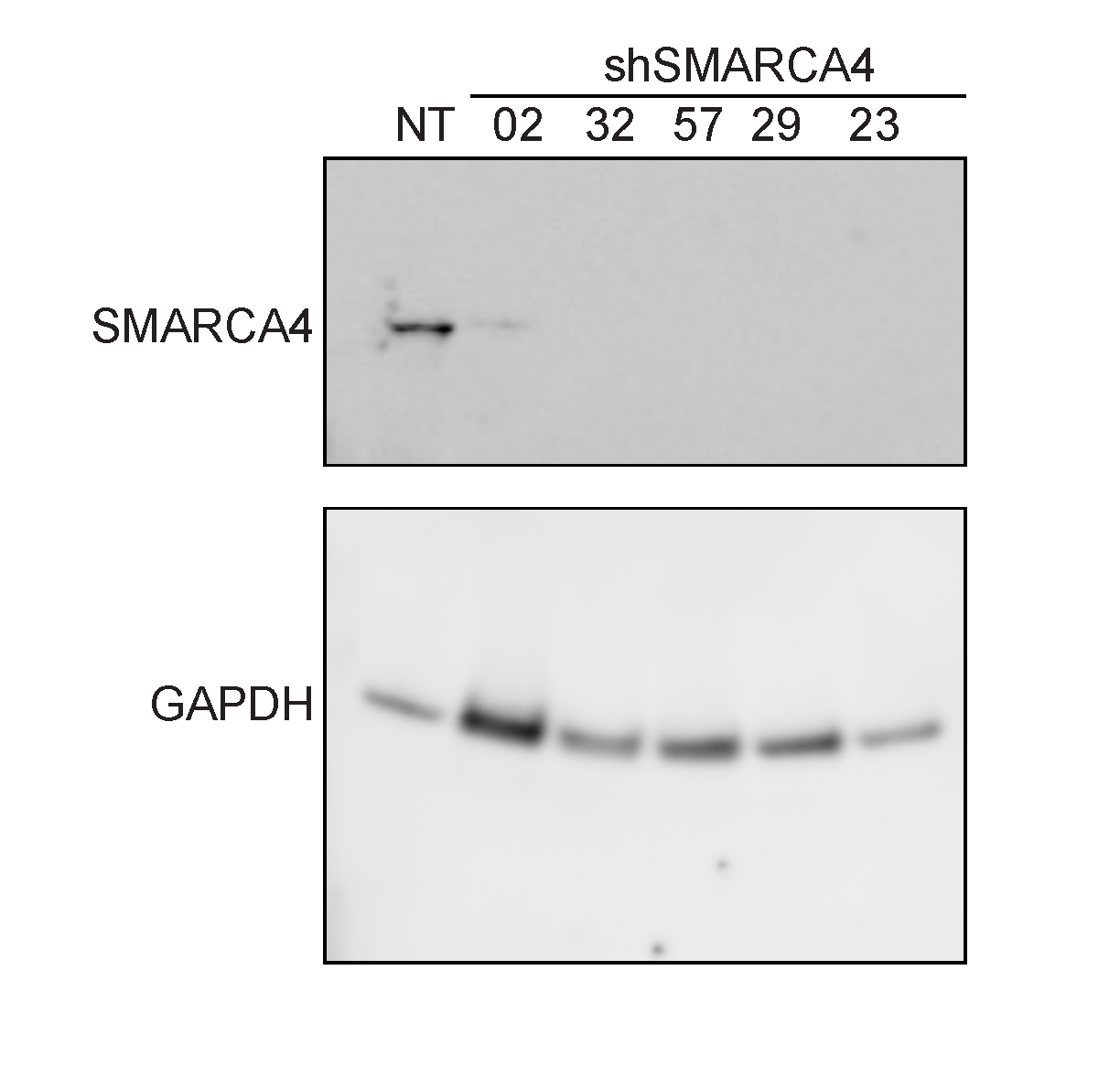

Supplement: S8 Fig — (TIFF) [file pone.0200826.s008.tiff]
